# Editorial: Sepsis: Basic, Clinical and Therapeutic Approaches

**DOI:** 10.3389/fphar.2022.910332

**Published:** 2022-05-11

**Authors:** Yan Kang, Daolin Tang, Lefu Lan, Hong Zhou

**Affiliations:** ^1^ Department of Critical Care Medicine, Institute of Critical Care Medicine, West China Hospital, Sichuan University, Chengdu, China; ^2^ West China Tianfu Hospital, Sichuan Universities, Chengdu, China; ^3^ Department of Surgery, UT Southwestern Medical Center, Dallas, TX, United States; ^4^ Shanghai Institute of Materia Medica, Chinese Academy of Sciences, Pudong Zhangjiang Hi-Tech Park, Shanghai, China; ^5^ Key Laboratory of Basic Pharmacology of Ministry of Education and Joint International Research Laboratory of Ethnomedicine of Ministry of Education, Zunyi Medical University, Zunyi, China

**Keywords:** sepsis, immune disorder, drugs, drug target, re-evaluation

Sepsis is a life-threatening organ dysfunction that affects millions of people globally; between one in six and one in three sepsis patients die each year (Evans et al., 2021). Its pathogenesis is complex and involves multiple systems and organs, and it requires comprehensive management. In particular, the immune system plays a core role through an early cytokine storm and late immunosuppression. However, no specific drug can effectively balance the uncontrolled immune reaction associated with sepsis (Evans et al., 2021). The Research Topic of this editorial focuses the development of new drugs and drug targets to combat sepsis. We believe that the research papers described below provide a new direction for investigations into drugs for the treatment of sepsis.

Autophagy is a conserved cellular biological process that plays an important role in maintaining the immune response, and is considered a new target for sepsis treatment (Ren et al., 2017). Liu et al. have described the role of transcription factor EB (TFEB) in the autophagy-lysosomal pathway. They found that some TFEB activators upregulated the mRNA expression of TFEB and, thereby, led to the activation of autophagy-lysosomal pathway (ALP) and promotion of the cellular clearance machinery.

Pathogen-associated molecular patterns, such as endotoxin/lipopolysaccharide (LPS), are important molecules associated with sepsis (van der Poll, et al., 2021). Qiao et al. demonstrated that capsaicin protects cardiomyocytes against LPS-induced damage via augmentation of 14-3-3γ-mediated autophagy. In the realm of herbal drugs, Wang et al. reported the effect of Xuebijing (XBJ), which is an add-on treatment that has been used for sepsis management for over 15 years in China. XBJ specifically targets sepsis-induced myocardial dysfunction, and it was found to significantly improve the survival of cecal ligation and puncture (CLP)-induced sepsis model mice by alleviating cardiac dysfunction. Its therapeutic effect was thought to be mediated by the combined action of its components paeoniflorin and hydroxysafflor yellow A.

Exosomes are vesicles derived from double invagination of the plasma membrane and are closely associated with immune responses. The cargos delivered by exosomes into recipient cells, especially immune cells, effectively alter their response and functions in sepsis (Murao et al., 2020). Qiu et al. investigated the effects and mechanisms of exosomes of multiple immune cells and the role of immune cell-derived exosomes in sepsis and provided an in-depth understanding of the mechanism of immune dysfunction in sepsis. Additionally, Murao et al. showed that exosomes transport cold-inducible RNA-binding protein (CIRP) from the intracellular to extracellular space in LPS- and CLP-induced sepsis mouse models. Thus, targeting exosome-mediated CIRP release may be a potential strategy for sepsis treatment.

Sepsis is often accompanied by metabolic disorders such as stress hyperglycemia, which damages host immune response and increases the risk of organ damage (Ali et al., 2008). Accordingly, the levels of endogenous glucagon-like peptide-1 (GLP-1) are tightly associated with the mortality of sepsis patients (Donnelly, 2012). In addition, Yang et al. reviewed the possible application of GLP-1 receptor agonists (GLP-1RAs), a new drug in diabetes treatment, for the treatment of sepsis. They suggested that GLP-1RAs not only regulated blood glucose homeostasis but also improved organ dysfunction, regulated immunity, and controlled inflammation and other functions (such as renal function) in sepsis. Their study provides a new perspective treatment for sepsis patients with stress hyperglycemia.

Epigenetic regulation plays an important role in the pathogenesis of sepsis (Falcão-Holanda et al., 2021). The epigenetic changes in sepsis include DNA methylation, histone modifications, and regulation of transcription via non-coding RNAs (Zhang et al., 2019). The bromodomain and extra-terminal (BET) protein family, an epigenetic regulator of gene transcription, has recently been recognized as a significant regulator of inflammation and immune response in sepsis. Wang et al. opined that BETs not only function as scaffolds to recruit different transcription factors and transcription elongation complexes, but also serve as switches to initiate gene transcription machinery in response to the interaction of BDs with acetylated chromatin either at gene promoters or in long-range cis regulatory elements. This mini-review summarizes the emerging roles and applications of BETs in sepsis.

Since 2019, the COVID-19 pandemic has been a threat to public health and killed millions of people worldwide (Alhazzani et al., 2021). In the early days of COVID-19, anti-pro-inflammatory cytokine antibodies were considered a promising treatment based on the observation that the over-release of pro-inflammatory cytokines was the cause of cytokine storms during COVID-19 (Schultze and Aschenbrenner, 2021). Accordingly, the systematic review by Wang et al. showed that the anti-cytokine antibodies tocilizumab, sarilumab, and anakinra could reduce mortality in patients with COVID-19; in particular, tocilizumab was not significantly associated with any serious adverse events or secondary infections. Further, Yuan et al. have also reviewed the potential of immune-related therapy in COVID-19 treatment and shed light on the current status of and advances in immune-related therapy for COVID-19 worldwide. In contrast to the promising effect of anti-cytokine therapy in COVID-19 treatment, in sepsis treatment, many clinical studies have found that antibodies have no therapeutic effect and may even increase mortality (Alhazzani et al., 2021). Thus, the use of anti-cytokine antibodies in the treatment of sepsis is controversial.

Treating sepsis with a combination of vitamins (such as vitamin C and vitamin D) and probiotics is currently an area of great interest. Kamel et al. demonstrated that both combinations of interventions, vitamin C plus vitamin B1 and vitamin D plus oral Lactobacillus probiotics, improved APACHE II scores and reduced sepsis incidence in trauma patients. Further, in their randomized, double-blinded, controlled clinical trial, severe trauma patients were stratified by leukocyte anti-sedimentation rate (LAR) into high-risk and low-risk groups for sepsis, and LAR combined with injury severity score was found to be a good predictor of sepsis.

In summary, the articles described here report on novel targets in immunotherapy and new therapeutic approaches in sepsis treatment. However, more research is required on new drug targets based on the pathophysiological mechanism of sepsis and re-evaluation of the data on existing drugs in clinical trials.
